# Aging Predisposes Oocytes to Meiotic Nondisjunction When the Cohesin Subunit SMC1 Is Reduced

**DOI:** 10.1371/journal.pgen.1000263

**Published:** 2008-11-14

**Authors:** Vijayalakshmi V. Subramanian, Sharon E. Bickel

**Affiliations:** Department of Biological Sciences, Dartmouth College, Hanover, New Hampshire, United States of America; Stowers Institute for Medical Research, United States of America

## Abstract

In humans, meiotic chromosome segregation errors increase dramatically as women age, but the molecular defects responsible are largely unknown. Cohesion along the arms of meiotic sister chromatids provides an evolutionarily conserved mechanism to keep recombinant chromosomes associated until anaphase I. One attractive hypothesis to explain age-dependent nondisjunction (NDJ) is that loss of cohesion over time causes recombinant homologues to dissociate prematurely and segregate randomly during the first meiotic division. Using Drosophila as a model system, we have tested this hypothesis and observe a significant increase in meiosis I NDJ in experimentally aged Drosophila oocytes when the cohesin protein SMC1 is reduced. Our finding that missegregation of recombinant homologues increases with age supports the model that chiasmata are destabilized by gradual loss of cohesion over time. Moreover, the stage at which Drosophila oocytes are most vulnerable to age-related defects is analogous to that at which human oocytes remain arrested for decades. Our data provide the first demonstration in any organism that, when meiotic cohesion begins intact, the aging process can weaken it sufficiently and cause missegregation of recombinant chromosomes. One major advantage of these studies is that we have reduced but not eliminated the SMC1 subunit. Therefore, we have been able to investigate how aging affects normal meiotic cohesion. Our findings that recombinant chromosomes are at highest risk for loss of chiasmata during diplotene argue that human oocytes are most vulnerable to age-induced loss of meiotic cohesion at the stage at which they remain arrested for several years.

## Author Summary

In humans, chromosome segregation errors during meiosis are the leading cause of birth defects and miscarriages. Moreover, as women age, these errors increase dramatically. For accurate segregation during the first meiotic division, homologous chromosomes must remain physically associated until anaphase I. Normally, attachments along the arms of sister chromatids keep the recombinant homologues together. Human oocytes complete meiotic recombination during fetal development and arrest until ovulation. Therefore, accurate segregation of homologous chromosomes during the first meiotic division requires that recombinant chromosomes remain associated for decades. One hypothesis to explain why segregation errors increase as women age is that the connections between sister chromatids deteriorate over time and allow recombinant homologues to dissociate prematurely. Here, we address this hypothesis using Drosophila as a model system. We find that when Drosophila oocytes undergo experimentally induced aging, recombinant homologues missegregate during meiosis I. Furthermore, the meiotic stage at which Drosophila oocytes are most vulnerable to age-induced errors is analogous to the stage at which human oocytes remain arrested for decades. Together, our data argue that aging does cause premature loss of the connections between meiotic chromosomes and that this is a major determinant of segregation errors in both Drosophila and human oocytes.

## Introduction

In humans, meiotic chromosome segregation errors that give rise to aneuploid gametes are the leading cause of fetal loss and birth defects [1]. Approximately 30% of miscarriages result from aneuploidy and at least 5% of all clinically recognized pregnancies and 0.3% of live-borns are aneuploid [1]. Female meiosis in humans is especially error-prone and the majority of segregation errors in oocytes originate during meiosis I [1],[2].

The link between increased maternal age and meiotic segregation defects in humans is well established. At maternal age 25, the risk of a trisomic pregnancy is approximately 2% but increases to approximately 35% for a woman at age 42 [3]. Despite its clinical importance, the specific mechanisms that give rise to age-dependent meiotic nondisjunction (NDJ) are not understood. The prevailing theory is that segregation errors in older oocytes arise in large part because of the protracted prophase I arrest at which human oocytes remain suspended for decades [1],[4].

**Figure 1:**
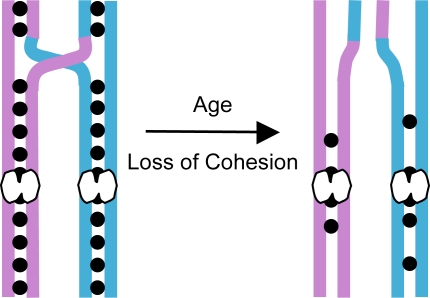
Proposed mechanism for age-dependent loss of chiasma maintenance in human oocytes. Different colors are used to represent the homologous chromosomes, each composed of identical sister chromatids. Meiotic cohesion is represented by solid black circles linking the sister chromatids. Centromeres are depicted as white bi-lobed structures. Sister chromatid cohesion distal to the site of a crossover is necessary to hold recombinant homologues together and this linkage ensures their accurate segregation during meiosis I. During meiotic prophase I, cohesion-dependent association of recombinant homologues is manifest as chiasmata that occur at crossover sites. Because meiotic recombination in human oocytes occurs during fetal development and is followed by a prolonged dictyate arrest, chiasma maintenance requires that sister cohesion remain intact for decades. One explanation for why meiotic segregation errors are more prevalent in older women is that cohesion between sister chromatids deteriorates with age and renders recombinant chromosomes susceptible to missegregation.

Accurate segregation during meiosis I requires that homologous chromosomes undergo recombination and remain physically attached to one another until they segregate to opposite poles during anaphase I. In the absence of a stable connection, homologues will segregate randomly resulting in meiosis I NDJ. Cohesion between the arms of sister chromatids provides an evolutionarily conserved mechanism for maintaining the association of recombinant homologues (Figure 1). Normally, the release of arm cohesion at anaphase I allows recombinant homologues to segregrate to opposite poles [5],[6]. In the absence of cohesion, chiasmata are not maintained and homologous chromosomes missegregate during meiosis I [5],[7],[8].

Given that human oocytes undergo meiotic recombination during fetal development and remain suspended in a prolonged dictyate (diplotene) arrest until ovulation, the continuous association of homologous chromosomes demands that meiotic sister-chromatid cohesion remain intact for decades. One attractive hypothesis to explain age-dependent NDJ is that deterioration of cohesion with age causes recombinant homologues to dissociate prematurely and segregate randomly during the first meiotic division (Figure 1). However, testing this hypothesis in humans presents several insurmountable challenges.

**Figure 2:**
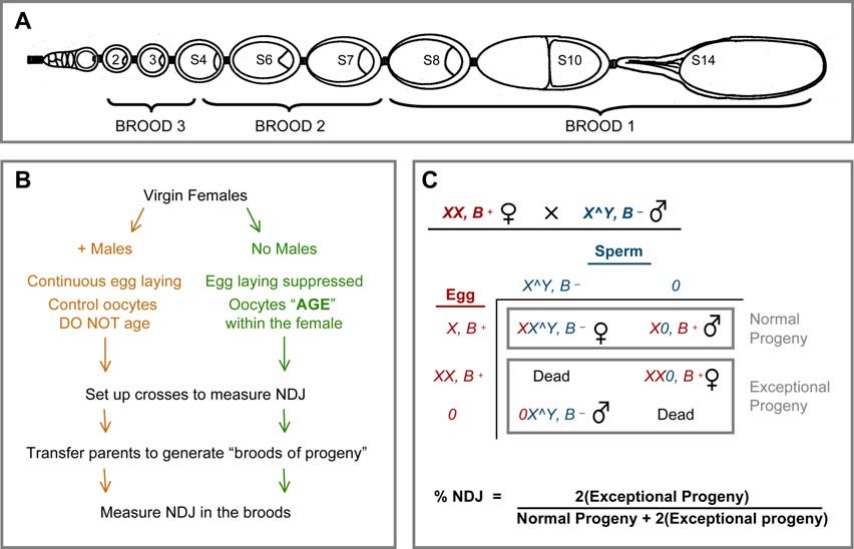
Scheme for aging Drosophila oocytes and measuring nondisjunction. (A) Each Drosophila ovary is composed of several ovarioles that contain a linear array of oocytes at progressive stages of development. Meiosis is initiated at the anterior tip of the ovariole, in the germarium (left). After exit from the germarium, the oocytes move posteriorly through the ovariole (stages 2 through 14). Not all oocyte stages are present at any given time in a single ovariole. This schematic is adapted from Robinson *et al.* [45]. (B) For the aging regimen, virgin females of the same genotype are divided into two groups. Control females (left) are incubated with males and lay eggs continuously. Experimental females (right) are deprived of males and egg-laying is suppressed. In these females, developmental progression of oogenesis is halted and oocytes “age” within the female. After four days of this aging regimen, control and experimental females are placed in vials with males and the flies are transferred at 24-hour intervals to generate “24 hour broods” of progeny for NDJ analysis. Each brood corresponds to oocytes that were halted at specific stages of development during the aging regimen (see panel A). Meiotic NDJ is measured in all three broods of both aged and non-aged oocytes (see below). (C) To measure NDJ, we made use of an *X* chromosome dominant marker, *Bar* (*B^−^*), that affects eye shape. *Bar^+^* experimental females are crossed to *Bar^−^* males that contain an attached *X∧Y* chromosome. The sperm generated by these males will contain either *X∧Y* with the *Bar^−^* marker or no sex chromosome (designated “*0*”). If meiotic segregation is normal in the female, oocytes will be produced that contain one *X* chromosome. However, segregation errors during female meiosis will result in exceptional gametes that contain either two *X* chromosomes (Diplo) or no *X* chromosomes (Nullo). The adult progeny resulting from normal and exceptional gametes can be distinguished based on their sex and eye shape (due to *Bar^−^*). In this test, all of the normal progeny survive, but only half of the exceptional progeny are viable. Therefore, the number of exceptional progeny is doubled and the total number of progeny is adjusted when calculating the %NDJ.

Using Drosophila as a model system, we have developed an experimental regimen to study the mechanisms that contribute to increased levels of meiotic NDJ in oocytes as a result of aging [9]. The Drosophila ovary is composed of several ovarioles, each of which contains a linear array of oocytes at progressive stages of development (Figure 2A). Throughout the lifetime of the female, germline stem cells at the tip of each ovariole continuously generate a steady stream of newly formed oocytes that enter meiotic prophase and grow and develop as they move posteriorly; as mature oocytes pass through the oviduct they complete meiosis and are fertilized. Under normal conditions (continuous egg laying), Drosophila oogenesis is an uninterrupted process with only a brief arrest at metaphase I before ovulation. However, when egg laying is suppressed, the majority of Drosophila oocytes within each ovariole are halted in developmental progression and “age” within the abdomen of the female (Figure 2B). Such experimentally induced aging of Drosophila oocytes can be used to mimic the normal aging process that human oocytes undergo within the ovary during a female's lifespan [9]. We have used this aging regimen to test the hypothesis that meiotic cohesion deteriorates as the oocyte ages and increases the frequency at which recombinant homologues missegregate. Although maternal age does not dictate the age of the oocyte in fruit flies as it does in humans, for simplicity we will refer to increased NDJ in experimentally aged Drosophila oocytes as “age-dependent NDJ.”

## Results

**Figure 3:**
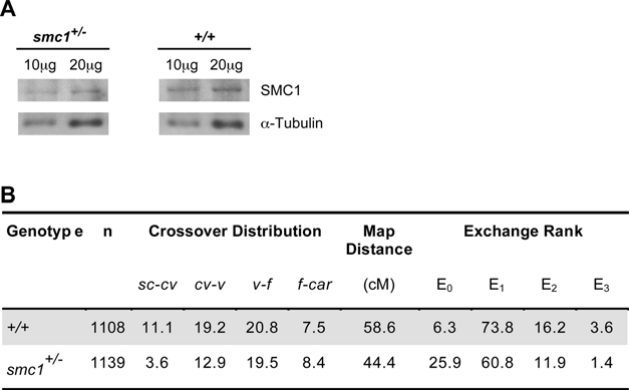
Chiasma formation is not severely disrupted when SMC1 is reduced. (A) Whole ovary extracts from wild-type and *smc1^+/−^* females were analyzed by immunoblot analysis using SMC1 antiserum [11]. α-tubulin was used as a loading control. Note that the relative intensity of SMC1 signal is lower in the *smc1^+/−^* extract than in wild type. A visual comparison suggests that SMC1 is reduced by approximately two-fold (SMC1 signal intensity is similar in the lanes containing 20 µg of *smc1^+/−^* extract and 10 µg of wild-type extract). (B) Meiotic recombination in wild-type and *smc1^+/−^* females was measured in four intervals along the *X* chromosome: *sc-cv*, *cv-v*, *v-f*, *f-car*. Crossovers were reduced in the distal intervals (*sc-cv*, *cv-v*) in *smc1^+/−^* oocytes. The tetrad exchange rank was computed from the recombination data and the percentage of bivalents for each rank is shown. Note that E_0_ tetrads increase in the mutant females at the expense of E_1_, E_2_, E_3_ tetrads.

The cohesin complex consists of four subunits and is required for sister-chromatid cohesion during mitosis and meiosis [6],[10]. Although wild-type Drosophila oocytes subjected to our standard four-day aging regimen do not exhibit increased levels of NDJ [9], we reasoned that this time period may be insufficient for cohesion to deteriorate enough to detect a loss in chiasma maintenance and that reduction of functional cohesin might render oocytes more vulnerable to aging effects. Unlike other eukaryotes, meiosis-specific cohesin subunits have not been uncovered in the Drosophila genome [11]. Therefore, to weaken but not eliminate meiotic cohesion, we reduced the dosage of the cohesin subunit SMC1 by utilizing flies heterozygous for a deletion that removes the *smc1* gene [12]. Immunoblot analysis confirmed that the level of SMC1 protein is reduced approximately two-fold in ovaries from *smc1^+/−^* females (Figure 3A).

### Reduction of SMC1 Protein Does Not Eliminate Crossovers

In order to assay loss of chiasma maintenance with age, we needed to verify that chiasma formation was not severely disrupted in *smc1^+/−^* oocytes. Cohesion between sister chromatids is required for normal levels of crossovers during meiosis [13]–[17]. Therefore, we measured the frequency and distribution of exchange in *smc1^+/−^* heterozygous females (Figure 3B). Although reduction of SMC1 protein has a semi-dominant effect on the number and distribution of meiotic crossovers during female meiosis, high levels of homologous exchange were still observed (76% of the wild-type control, Figure 3B). The tetrad exchange rank for Drosophila oocyte bivalents can be estimated from analysis of recombinant and non-recombinant meiotic products [18]. Normally, the *X* chromosome is achiasmate (non-recombinant) in 6–12% of Drosophila oocytes [19]. Our recombination data indicates that although crossovers were reduced in *smc1^+/−^* oocytes, the majority of tetrads (74%) had at least one exchange event (Figure 3B). In addition, viability and fertility of *smc1^+/−^* females is normal and they do not exhibit meiotic segregation defects in the absence of an aging regimen (Table S1). Therefore, *smc1^+/−^* oocytes provide an excellent sensitized system that can be used to assay whether cohesion and chiasma maintenance decline with age.

### Reduced Cohesin Leads to Age-Dependent NDJ

**Figure 4:**
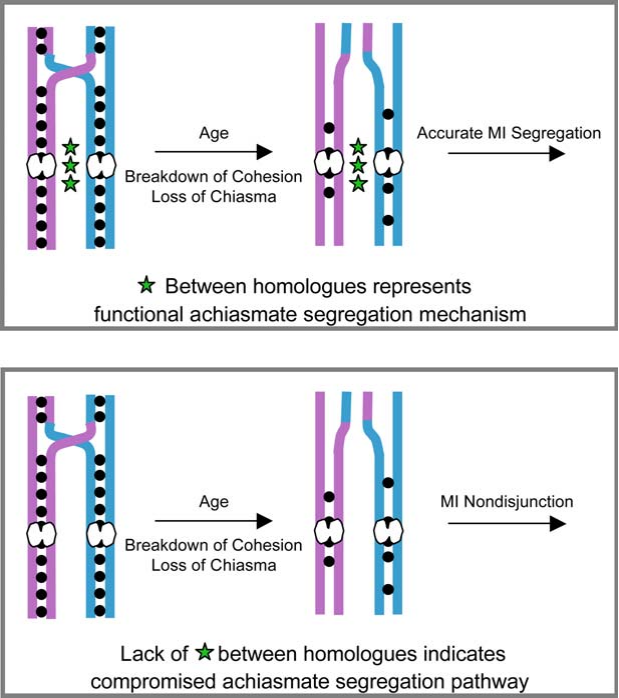
Scheme to assay loss of chiasmata with age in Drosophila oocytes. In the absence of crossovers, accurate segregation of bivalents in Drosophila oocytes is governed by the achiasmate pathway which relies on pericentric heterochromatin. In the presence of a functional achiasmate system, bivalents that lose their chiasmata will still segregate properly. Therefore, the achiasmate pathway must be compromised to determine whether chiasmata are lost as Drosophila oocytes age. Top panel: A functional achiasmate segregation system (depicted by green stars between homologues) will ensure accurate segregation of recombinant bivalents that have lost chiasmata during the aging process. Bottom panel: When the achiasmate pathway is compromised (depicted by the absence of green stars), recombinant bivalents that have lost chiasmata should missegregate.

To examine the effect of SMC1 dosage on age-dependent NDJ, we subjected *smc1^+/−^* females to the aging regimen and assayed for chromosome missegregation (see Figure 2B, 2C). However, meiotic NDJ did not increase when *smc1^+/−^* oocytes were aged (Table S2). Because Drosophila females harbor a robust mechanism that directs the segregation of achiasmate chromosomes [20], it is possible that this achiasmate mechanism also ensures accurate disjunction of recombinant chromosomes that fail to maintain chiasmata (Figure 4). Therefore, we reasoned that in order to detect missegregation of recombinant chromosomes in Drosophila oocytes that have lost chiasmata, the achiasmate system must be compromised (Figure 4). Hawley and colleagues have shown that P-element disruption of one copy of *matrimony* (*mtrm^[KG08051]/+^*) disrupts the segregation of achiasmate chromosomes in Drosophila oocytes [21]. However, meiotic cohesion, synaptonemal complex assembly and crossover frequency appear to be unaffected in *mtrm^+/−^* oocytes [21],[22]. Therefore, to monitor whether exchange bivalents lose chiasmata with age, we compromised the achiasmate pathway by reducing the dosage of *mtrm* in *smc1^+/−^* females.

**Figure 5:**
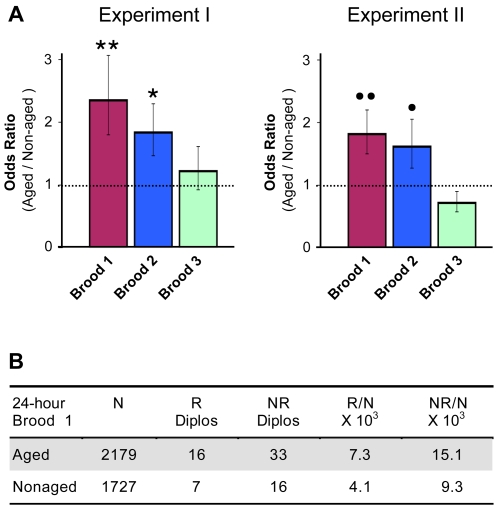
Both chiasmate and achiasmate homologues exhibit age-dependent NDJ in *smc1^+/−^ mtrm^+/−^* oocytes. (A) An odds ratio is used to represent the relative level of NDJ in aged versus non-aged oocytes for 24-hour broods. An odds ratio >1 indicates that NDJ is more likely in the aged oocytes than in the non-aged oocytes. The error bars correspond to ±1 standard error. Two independent experiments are shown. In both experiments, NDJ in aged oocytes is significantly higher than in non-aged oocytes for broods 1 and 2 (**P = 0.0010, *P = 0.0062; ••*P* = 0.0017, •*P* = 0.0439) but age-dependent NDJ is not observed for Brood 3 (*P* = 0.4751, *P* = 0.1416 for Experiments I and II respectively). Although the absolute levels of NDJ vary, the same pattern is observed for both experiments. For each experiment, at least 1000 progeny were scored for each brood. The raw data for the two independent experiments are shown in Tables S3A and S3B. (B) Diplo-*X* females from brood 1 of Experiment II were genotyped (see Figure 6). R denotes Diplo-*X* females containing at least one recombinant *X* chromosome. NR denotes Diplo-*X* females in which both *X* chromosomes were non-recombinant (as judged by the markers scored). N is the number of total progeny scored in the NDJ assay. The frequency at which R and NR chromosomes missegregate also is shown (R/N and NR/N). Missegregation of both recombinant and non-recombinant chromosomes is more prevalent in aged oocytes.

We observed increased segregation errors in *smc1^+/−^* oocytes following the four-day aging regimen when the achiasmate pathway was also compromised (*mtrm^+/−^*) (Figure 5A). Significant levels of age-dependent nondisjunction were observed for broods 1 and 2, which represent oocytes that are developmentally most mature during the aging regimen (see Figure 2A). We have performed multiple experiments to compare NDJ in *smc1^+/−^ mtrm^+/−^* aged and non-aged oocytes and have also assayed missegregation of different *X* chromosomes (data not shown). Although the absolute level of NDJ may vary between experiments, we have repeatedly observed higher levels of NDJ in *smc1^+/−^ mtrm^+/−^* aged oocytes in the first two 24 hour broods (see Tables S3A and S3B for examples of raw data from two independent experiments). In contrast, *mtrm^+/−^* oocytes with normal levels of SMC1 do not exhibit age-dependent NDJ in the first brood (Table S4); we did detect a significant increase in brood 2, but the absolute level of NDJ was relatively low (Table S4). The finding that increased meiotic chromosome missegregation in aged Drosophila oocytes occurs when the dosage of a cohesin subunit is reduced is consistent with deterioration of cohesion and loss of chiasma maintenance as an underlying cause for age-dependent NDJ.

### Recombinant Chromosomes Missegregate in Older *smc1^+/−^ mtrm^+/−^* Oocytes

**Figure 6:**
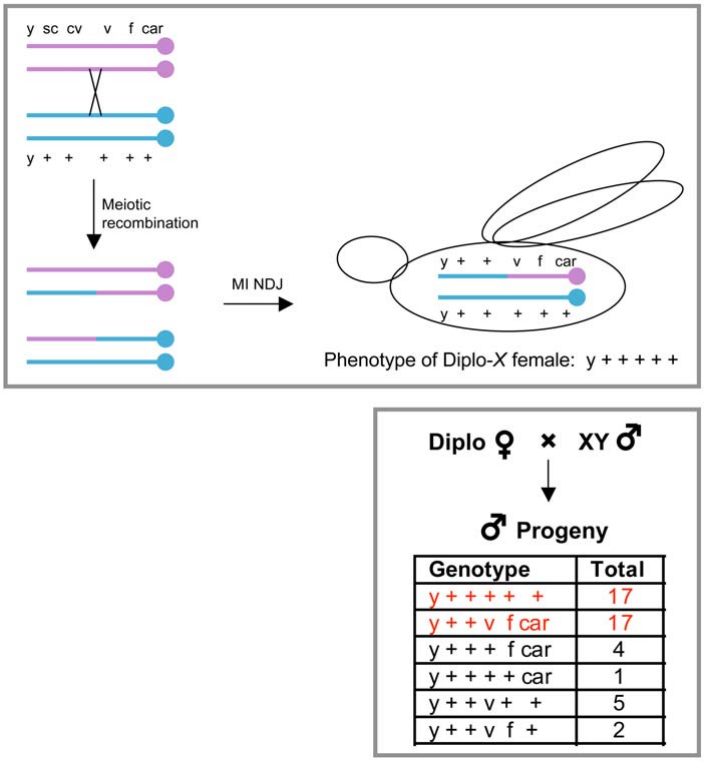
Scheme to determine the recombinational history of *X* chromosomes that missegregate in the NDJ test. A hypothetical example is presented in the two panels to illustrate how we determine the genotype of the two missegregating *X* chromosomes that give rise to a Diplo-*X* female. Top panel: In this scenario, a single crossover between *cv*-*v* on the *X* chromosome in the oocyte generates a bivalent with two recombinant and two non-recombinant chromatids. Missegregation during meiosis I results in an egg with two *X* chromosomes that is fertilized and develops into a “Diplo-*X*” adult female. In this schematic, the Diplo-*X* female inherited one recombinant and one non-recombinant *X* chromosome from the oocyte. Bottom panel: The Diplo-*X* female is crossed to a normal male and her male progeny are scored for the different markers on the *X* chromosome. Genotyping is complicated by the fact that crossovers can occur between the two *X* chromosomes in the oocytes of the Diplo-*X* females. However, the genotype of the *X* chromosomes in the Diplo-*X* female (right, top panel) can be assigned because in any recombination analysis, the parental chromosomes are the most predominant. In this example, the parental chromosomes are shown in red. Note that it is possible for a Diplo-*X* female to inherit two non-recombinant chromatids from a recombinant tetrad. In addition, a double crossover between two adjacent markers will be invisible. For these reasons, the frequency at which recombinant chromosomes nondisjoin is underestimated in our analyses.

To ask whether deterioration of chiasmata contributed to the increase in age-dependent nondisjuntion in *smc1^+/−^ mtrm^+/−^* oocytes, we assayed the recombinational history of the missegregating chromosomes. For this analysis, we collected “Diplo-*X*” female progeny that received two maternally contributed *X* chromosomes due to meiotic missegregation (see Figure 2C) and performed an additional cross to genotype the two *X* chromosomes (Figure 6). Using this strategy, we determined whether missegregating chromosomes had undergone one or more crossovers (Table S5). In addition, a centromere-proximal marker (*car*) allowed us to assess whether NDJ events involved homologous chromosomes (MI NDJ) or sister chromatids (MII NDJ). The genotype of the Diplo-*X* females suggested that the majority of NDJ events (70/72) occurred during meiosis I (Table S5). Approximately one-third of all Diplo-*X* females arose from nondisjunction of exchange tetrads (R) in both aged and non-aged oocytes (Figure 5B). Notably, recombinant bivalents missegregated 1.8 times more frequently in the aged oocytes than in non-aged oocytes (compare R/N for aged and non-aged, Figure 5B). In addition, the number of recombinant bivalents that missegregate is under-represented in our assay because it is possible for a Diplo-*X* female to inherit two non-recombinant chromatids from a recombinant tetrad (see Figure 6). These data argue that upon reduction of SMC1, recombinant bivalents become more vulnerable to missegregation when oocytes age.

Although the achiasmate pathway is compromised in *mtrm^+/−^* mutants, our data indicate that it is not completely disengaged. An estimated 26% of the tetrads are achiasmate in *smc1^+/−^* oocytes (Figure 3B) but we only observe 4–9% NDJ in non-aged oocytes when the achiasmate pathway is compromised (*smc1^+/−^ mtrm^+/−^*, Table S3). Moreover, segregation of an *X* chromosome (*In(1)dl-49*) harboring an inversion that severely suppresses crossovers is not completely random in *mtrm^+/−^* oocytes (Table S1). The data in Figure 5B indicate that when the level of SMC1 protein is reduced, aging further increases the frequency at which achiasmate chromosomes missegregate in *mtrm^+/−^* oocytes (compare NR/N for aged and non-aged). These data implicate the cohesin complex in accurate disjunction of achiasmate as well as chiasmate bivalents. However, the low number of MII NDJ events that we observe (as determined by the centromere proximal *car* marker) argues that the increased missegregation of non-recombinant chromosomes is not due to loss of centromeric cohesion between sister chromatids.

### Oocytes Are Most Susceptible to Age-Related Segregation Defects After Pachytene but Before Metaphase I

Our analysis of age-dependent NDJ in *smc1^+/−^ mtrm^+/−^* oocytes indicates that broods 1 and 2 are most susceptible to age effects. Each Drosophila ovariole consists of multiple oocytes at progressive stages of development. At the posterior end of the ovariole (stages 13/14), the mature oocytes undergo nuclear envelope breakdown and spindle assembly. Stage 14 oocytes remain arrested at metaphase I until passage through the oviduct triggers resumption of the meiotic divisions. After an aging regimen, the most mature oocytes are laid first and therefore will contribute to the progeny in brood 1 (see Figure 2A).

In mature oocytes, the chromosomes have already made stable connections with the meiotic spindle microtubules and premature loss of chiasmata during the aging regimen should not affect their segregation. However, if the mature oocytes develop gross defects in the spindle machinery as they age, recombinant chromosomes could missegregate by a mechanism unrelated to chiasma maintenance. Conversely, oocytes that undergo aging at a stage prior to meiotic spindle assembly would depend on chiasma maintenance for proper segregation. Therefore, it was important to determine what fraction of broods 1 and 2 correspond to metaphase I arrested oocytes.

**Figure 7:**
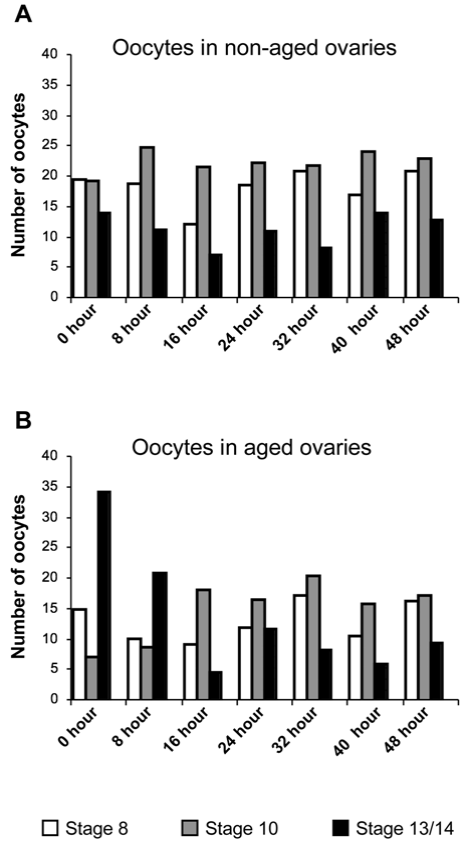
Quantification of oocyte stages present in non-aged and aged ovaries at different time-points following the aging regimen. The number of oocytes at each of the indicated stages was tabulated for one ovary from three different *smc1^+/−^ mtrm^+/−^* females and the average is presented. Immediately after the aging regimen (time-point 0), the number of mature oocytes (stage 13/14) is ∼2.5 fold higher in the aged ovaries than in non-aged ovaries. This contrasts sharply with the decline in stage 10 oocytes at this same time point. The number of stage 8 oocytes is similar in aged and non-aged ovarioles immediately following aging regimen. These results are consistent with a previous report [23] that when egg-laying is inhibited, stages 1–8 halt in development and “age” while stages 9–13 continue to develop and only arrest once they reach stage 14. After egg-laying resumes, the relative abundance of each of the different stages is similar in aged and non-aged ovaries by 16 hours although fewer oocytes are present in the aged ovaries.

To compare the relative distribution of oocytes at different developmental stages in ovaries from *smc1^+/−^ mtrm^+/−^* control females and those subjected to the aging regimen, we examined ovaries at 8-hour intervals after completion of the four-day regimen. One striking difference was the increased number of mature oocytes (stages 13/14) following aging (Figure 7 and Figure S1). Previous reports by other investigators also have documented that mature oocytes accumulate when egg laying is suppressed [23],[24]. Quantitative analysis indicated that our aging regimen resulted in a ∼2.5-fold increase in mature oocytes compared to the ovarioles of control females (Figure 7). However, after 16 hours of egg laying, this excess of mature oocytes was no longer observed. These results argue that in our NDJ tests, at least some fraction of progeny in brood 1 (0–24 hours) arise from oocytes that have not yet assembled meiotic spindles.

Evaluation of the distribution of different developmental stages in fixed ovaries also indicated that following the aging regimen, the increase in mature oocytes occurred primarily at the expense of stage 9–12 oocytes (Figure 7) while oocytes at earlier stages (stages 1–8) remained abundant. Significantly, we found that 16 hours after the aging regimen ceased and egg laying commenced, the distribution of late stages in the aged group resembled that in control ovaries, although the total number of oocytes between stages 8 and 14 was slightly less in the aged ovaries. Consistent with our findings, King [23] reported that stages 9–12 are significantly under-represented in ovaries of 4 day old virgins. Together, these data indicate that oocytes at stages1–8 halt in development and age during the four-day aging regimen, while oocytes at later stages (stages 9–13) continue to mature and do not “age” until they arrest at metaphase I (stage 14).

Given the altered distribution of oocytes at different developmental stages following the aging regimen, we carried out NDJ tests to determine which stages during oocyte development are the most susceptible to age-dependent segregation errors. We subjected *smc1^+/−^ mtrm^+/−^* females to our standard four-day aging regimen and set up crosses to assay meiotic NDJ (see Figure 2). However, this time, we transferred the parents to new vials every 8 hours (instead of every 24 hours) to generate 8-hour “sub-broods” of progeny. This allowed us to measure age-related segregation errors in “sub-broods” of progeny that represented snapshots of oocytes at different meiotic stages (pachytene, diplotene-like and metaphase I).

**Figure 8:**
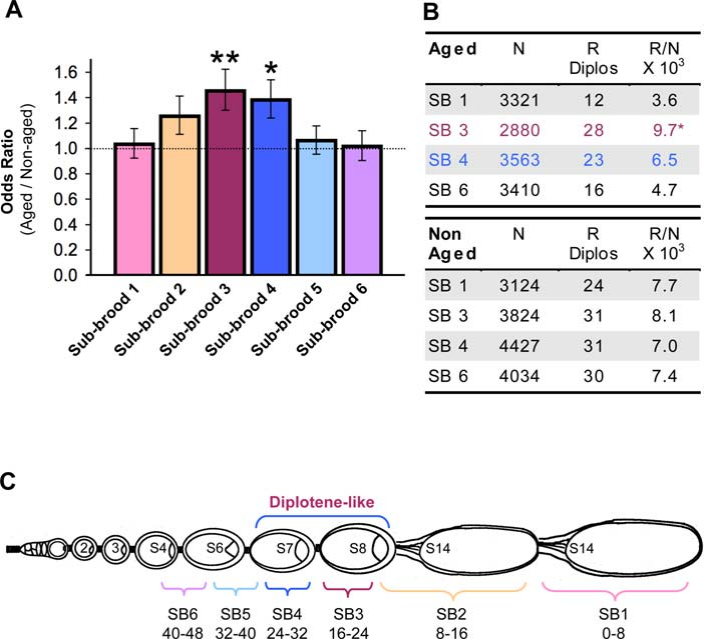
Diplotene-like oocytes are most vulnerable to age-dependent nondisjunction. (A) For each of the different 8-hour sub-broods indicated, the relative level of NDJ in aged versus non-aged *smc1^+/−^ mtrm^+/−^* oocytes is represented as an odds ratio. The error bars correspond to ±1 standard error. Oocytes laid between 16–32 hours after the aging regimen (sub-broods 3 and 4) exhibit significant age-dependent NDJ (**P = 0.0009 and *P = 0.0035). The data shown represent pooled data from two independent experiments in which at least 1200 progeny were scored for each sub-brood. The combined raw data are shown in Table S6. (B) For aged oocytes, the frequency at which recombinant chromosomes missegregate (R/N) in sub-brood 3 is significantly different from that for both sub-broods 1 and 6 (*P* = 0.0037, for sub-brood 3 versus 1, *P* = 0.022 for sub-brood 3 versus 6). In contrast, the frequency of R Diplos is similar in all four sub-broods analyzed from non-aged oocytes (0.73<*P*<0.92 for pair-wise comparisons). (C) Schematic of an ovariole following the aging regimen reflects the accumulation of metaphase I arrested oocytes (stage 14) that occurs at the expense of stages 9–13 (compare with Figure 2A). Unlike stages 9–13 which continue to progress, oocytes at stages 1–8 halt in development during the aging regimen. However, only oocytes that undergo aging during diplotene (stages 7–8) exhibit significant levels of age-dependent NDJ. In contrast, oocytes that remain arrested at metaphase I are not susceptible to age effects. Schematic is adapted from Robinson *et al.* [45].

Following the aging regimen, age-dependent NDJ was observed in sub-broods 3 and 4, which arose from oocytes that were fertilized 16–32 hours after the aging regimen was completed (Figure 8A, Table S6). Our cytological analysis (Figure 7 and Figure S1) and the published time-frame for oogenesis progression [19],[25], indicate that these sub-broods correspond to oocytes at stages 7 and 8 which have already disassembled their synaptonemal complex before initiation of the aging regimen [26],[27]. In contrast, a significant increase in meiotic segregation errors was not observed during the first 16 hours in aged *smc1^+/−^ mtrm^+/−^* oocytes (Figure 8A, Table S6). These results argue that metaphase I arrested *smc1^+/−^ mtrm^+/−^* oocytes (stage 14) are not vulnerable to age-related defects. Similarly, oocytes that undergo aging during pachytene (sub-broods 5 and 6) also appear to be refractory to age effects. Therefore, only oocytes that age during a very specific window of oogenesis (post-pachytene/pre-metaphase I) exhibit age dependent NDJ.

To determine what fraction of missegregating chromosomes were recombinant in the aged versus non-aged oocytes, we genotyped the Diplo-*X* progeny for a subset of sub-broods obtained from the NDJ analysis shown in Figure 8A. In addition to sub-broods 3 and 4 which exhibited age-dependent nondisjunction, we also analyzed the recombinational history of missegregating-*X* chromosomes in sub-broods 1 and 6, which represent opposite ends of the developmental spectrum examined in this study (Tables S7, S8). Again, we found that both recombinant and non-recombinant chromosomes missegregated in aged and non-aged oocytes (Tables S7, S8). In addition, a striking trend emerged for the aged oocytes when we calculated the frequency at which recombinant (R) chromosomes missegregated in each sub-brood (R/N; Figure 8B). NDJ of recombinant chromosomes in sub-brood 3 aged oocytes was significantly higher than that for aged oocytes in sub-brood 1 (*P* = 0.0029) or sub-brood 6 (*P* = 0.0179). In addition, this trend holds true when the NDJ of recombinant chromosomes in sub-brood 4 aged oocytes is compared to that for sub-broods 1 or 6 aged oocytes, although the difference is not statistically significant. These data contrast strongly with those for non-aged oocytes, in which recombinant chromosomes missegregate at similar frequencies in the four sub-broods analyzed.

The data shown in Figure 8 provide compelling evidence that only oocytes at a specific meiotic stage are vulnerable to age-dependent segregation errors even when all oocytes within the ovary are subjected to the aging regimen. The increased nondisjunction observed in specific sub-broods arises in part because segregation of recombinant bivalents becomes more error-prone (Figure 8B). When one takes into account the well-documented time-line for oocyte development while evaluating the sub-brood NDJ results, a striking pattern emerges (Figure 8C). When pachytene oocytes undergo the aging regimen, recombinant chromosomes do not exhibit an age-dependent increase in nondisjunction (sub-broods 5 and 6). In contrast, sub-broods 3 and 4, which display the greatest age-related defects, are composed primarily of oocytes at stages 7 and 8 of oocyte development. Both electron microscopy and immunofluorescence analyses have verified that disassembly of the synaptonemal complex in Drosophila oocytes initiates prior to these stages [26],[27]. Although Drosophila chromosomes do not exhibit typical diplotene/diakinesis morphology in prophase I oocytes, stages 7 and 8 of oogenesis correspond to these classical meiotic stages [19]. Transit through each of these stages takes approximately 5–8 hours during normal oogenesis, but when their developmental progression is halted by our aging regimen, meiotic chromosomes remain suspended in this state up to 19 times longer than usual. Of special note is the finding that recombinant chromosomes are most susceptible to segregation errors if Drosophila oocytes undergo aging during diplotene (Figure 8) because this is the meiotic stage at which human oocytes remain arrested for decades.

Our sub-brood analysis also suggests that non-recombinant chromosomes in *smc1^+/−^ mtrm^+/−^* oocytes missegregate more frequently when aging is induced in oocytes prior to metaphase I (Figure S2), although the difference between sub-brood 1 and the other sub-broods is not statistically significant (0.10<*P*<0.27). However, non-recombinant chromosomes in sub-brood 3 are significantly more vulnerable to missegregation after aging (*P* = 0.029). These results support the model that the connections that keep achiasmate bivalents together depend at least in part on meiotic cohesion proteins [9].

## Discussion

Together, our data support the hypothesis that weakening of meiotic cohesion with age is a significant determinant of age-dependent nondisjunction in both fly and human oocytes. One major advantage of these studies is that we have lowered but not eliminated the level of the cohesin subunit SMC1. Although *smc1^+/−^* oocytes are sensitized to the effects of aging, meiotic cohesion is initially intact. Therefore our regimen allows us to observe the effect of aging upon the normal cohesin complex that functions during Drosophila meiosis. In this respect our analysis differs markedly from previous studies using knock-out mice in which the meiosis-specific subunit SMC1-β was eliminated [8].

Here we demonstrate that deterioration of normal meiotic cohesion during the aging process causes loss of chiasma maintenance. Loss of arm cohesion in aged oocytes accounts for the increased frequency with which recombinant chromosomes missegregate during meiosis I. In contrast, low levels of MII NDJ indicate that centromeric cohesion remains intact, at least for the duration of our aging regimen. Recombinant chromosomes in Drosophila oocytes that undergo aging after they have disassembled their synaptonemal complex (diplotene-like) are most vulnerable to age effects and these oocytes bear a striking resemblance to the stage at which human oocytes remain arrested for decades. Interestingly, Drosophila oocytes that are subjected to aging during pachytene do not exhibit significant age-dependent nondisjunction. Using epifluorescence microscopy to compare *smc1^+/−^ mtrm^+/−^* aged and non-aged oocytes, we have not observed any obvious differences in SC morphology, or the timing of its assembly or disassembly (VVS and SEB, unpublished results). Therefore, we speculate that the synaptonemal complex may play a role in protecting sister-chromatid cohesion from age-induced deterioration. The differential response of pachytene and diplotene-like Drosophila oocytes to the aging process has profound implications regarding the vulnerability of human oocytes to age-induced loss of meiotic cohesion. Our data argue that the meiotic stage (dictyate) at which human oocytes remain suspended as women age is also the stage during which recombinant chromosomes are at highest risk for loss of chiasmata.

Because the achiasmate pathway in Drosophila oocytes will ensure the accurate segregation of recombinant chromosomes that lose chiasmata due to deterioration of arm cohesion during the aging process, we needed to dismantle this system for our analysis. Therefore, we used a P-element insertion in *matrimony* (*mtrm^KG08051^*) as a genetic tool to disrupt the achiasmate pathway. However, we do not think that heterozygosity for *mtrm* contributes to age-dependent loss of arm cohesion. Recent work has demonstrated that Mtrm protein physically interacts with and inhibits the activity of Polo kinase in Drosophila oocytes from stage 11 until nuclear envelope breakdown (NEB) at stage 13 [22]. When Mtrm protein is reduced (*mtrm^+/−^*), NEB can occur prematurely (at stage 12) and the spindle assembles around a karyosome that is less compact than in wild type. Although this may account for the eventual missegregation of achiasmate chromosomes in *mtrm^+/−^* heterozygotes, sister chromatid cohesion remains intact in these oocytes as evidenced by the striking chiasmata visible in the individualized bivalents [22]. In addition, premature NEB in *mtrm^+/−^* oocytes still occurs much later than the stages that exhibit sensitivity to aging in our experiments (stages 7–8). Therefore, we conclude that reducing the dosage of Mtrm is not causing the increased missegregation of recombinant chormosomes that we observe in aged oocytes.

Because the achiasmate segregation pathway is not completely disrupted in *mtrm^KG08051^* heterozygotes, we also have been able to assess the effect of aging on the segregation of non-recombinant chromosomes. Interestingly, our data demonstrate that when the dosage of SMC1 is reduced, MI missegregation of non-recombinant chromosomes also increases with age. This result is reminiscent of our previous findings that NDJ of achiasmate chromosomes increases with age when activity of the meiotic cohesion protein ORD is compromised by a hypomorphic missense mutation [9]. In Drosophila oocytes, accurate segregation of achiasmate chromosomes relies on homologous pairing of centromere proximal heterochromatin [28]–[30] and both ORD and the cohesin complex are highly enriched at the percentric heterochromatin in oocytes [11],[17]. In addition, our recent data indicate that heterochromatin-mediated pairing is moderately disrupted by *ord* mutations, even in the absence of aging (VVS and SEB, unpublished results). Together, these data suggest that in addition to holding sister chromatids together, cohesion proteins play an important role in maintaining the heterochromatic pairing of achiasmate homologues in Drosophila oocytes. Whether cohesion proteins function directly to “glue” homologues together or play a more indirect role, such as influencing the structure of heterochromatin, remains to be determined. Regardless of the mechanism, loss of heterochromatin-associated cohesion proteins with age could account for the increased missegregation of non-recombinant chromosomes that we observe.

Our data support the hypothesis that age-dependent NDJ in women is caused at least in part by progressive loss of sister-chromatid cohesion over time. One unresolved issue regarding meiotic cohesion in human oocytes is whether the original cohesin molecules used to establish cohesion are maintained for decades or continually replaced as oocytes age. Addressing this question will be essential in understanding the specific mechanisms that lead to loss of meiotic cohesion and chiasma maintenance during the aging process. Early experiments in yeast led to the widely accepted model that cohesion between sister chromatids can only be established during DNA replication [31],[32]. However, recent evidence in mammalian tissue culture cells indicates that cohesin association with chromatin is much more dynamic than originally predicted [33]. In addition, experiments in yeast have recently demonstrated that cohesion can be re-established on a genome-wide scale during G2/M in a DNA damage-dependent manner [34],[35] and support the model that non-canonical mechanisms can establish cohesion after S phase is completed.

The power of genetic manipulation combined with the ability to age oocytes makes Drosophila an ideal model system to address whether re-establishment of meiotic cohesion occurs during prophase I. These future studies will be pivotal in understanding how cohesion dynamics during meiosis govern chiasma maintenance and ultimately, why and how loss of cohesion occurs as oocytes age.

## Materials and Methods

### Drosophila stocks

Flies were reared at 25°C on standard cornmeal molasses media. The *smc1^ex46^* allele is a deletion that removes the gene [12] and is denoted as *smc1^−^*. The *mtrm^[KG08051]^* allele results from a P-element disruption of the gene [21] and is denoted as *mtrm^−^*. Descriptions of the other genetic markers and chromosomes used in this study can be found at http://www.flybase.org.

Although several proteins (Axs, Ald/Mps1, Nod and Mtrm) are required for the accurate segregation of achiasmate chromosomes in Drosophila oocytes, we considered a *mtrm* allele to be the ideal choice for our studies [21],[22]. In contrast to *nod*, achiasmate segregation is severely affected in the *mtrm* heterozygote and chromosome loss is not observed [36]. In addition, unlike *Axs* oocytes, spindle defects and premature onset of anaphase I have not been observed in *mtrm* heterozygotes [37]–[39]. We thought it unwise to compromise achiasmate segregation using *ald* mutations given the role of this protein in the spindle assembly checkpoint; *ald* oocytes also exhibit low levels of NDJ of the exchange bivalents [40]–[42]. The specificity of the mutant phenotype in *mtrm^+/−^* oocytes combined with the observation that sister-chromatid cohesion appears to be normal convinced us that this was the best approach to disrupt the achiasmate segregation pathway.

### Recombination Analysis

To assay *X*-chromosome crossover frequency and distribution in *smc1^+/−^* oocytes, 5–7 females were crossed to 3 *yw* males per vial. Crossover frequency and distribution was measured by assaying *sc*, *cv*, *v*, *f*, *car* markers in the male progeny. The recombination data was used to estimate tetrad exchange ranks [18].

### Aging Regimen and Generation of Broods

To age Drosophila oocytes, the aging regimen described by Jeffreys *et al.* was modified [9] such that the glucose agar media was prepared without the addition of fungal inhibitors (methyl paraben and ethyl acetate). The glucose agar media contained 2% agar (Fisher) and 5% dextrose (Fisher) and was prepared with milli-Q grade water. Yeast paste was prepared by dissolving 30 g of active dry yeast (Red Star) in 50 mL milli-Q grade water.

A schematic of the aging regimen is shown in Figure 2B. Approximately 200 virgin females of the desired genotype were collected within an 8-hour period and the females were fed yeast overnight in vials with cornmeal molasses media to promote vitellogenesis. Overnight incubation of females with yeast allows yolk deposition and maturation of oocytes so that the ovaries contain a complete complement of oocytes at the different stages. The following day, females were split into two groups and placed in separate plexi-glass laying bottles containing glucose agar plate with a smear of yeast paste. The control group of females was supplied with an equal number of male flies and laid their eggs continuously. Their oocytes were “non-aged”. The experimental group of females were deprived of males. Because oviposition was suppressed in these females and the developmental progression of oogenesis was halted, oocytes “aged” within their abdomens. This experimentally induced aging of oocytes mimics the aging of oocytes in human females. Control and experimental flies were held in the laying bottles for four days with fresh yeast paste/glucose-agar plates supplied each day.

### Generation of 24-Hour Broods of Progeny

At the end of the four-day aging regimen, the experimental females (with aged oocytes) and the control females (with non-aged oocytes) were crossed to *X∧Y*, *v f B* males to measure meiotic nondisjunction in the oocytes (see Figure 2C). To generate 24-hour broods, 7 female flies were mated with 3–5 *X∧Y*, *v f B* males (per vial). The parents were transferred to new vials every 24-hours and three broods of progeny were analyzed for NDJ.

### Generation of 8-Hour Sub-Broods of Progeny

For simplicity, we have used the term “sub-broods” to differentiate 8-hour broods of progeny from the 24-hour broods of progeny. To generate sub-broods of progeny at the end of the aging regimen, 21 experimental or control females were crossed to 10 *X∧Y*, *v f B* males (per vial). The parents were transferred to new vials every 8-hours for a total of 48 hours and six sub-broods of progeny were analyzed for NDJ.

### Nondisjunction Assay

Because Drosophila can tolerate certain sex chromosome aneuploidies, segregation errors during meiosis can be monitored in the viable progeny by using differentially marked sex chromosomes (see Figure 2C). To compensate for the fact that only half of the exceptional progeny survive (see Figure 2C), total NDJ was adjusted according to the following formula: [2*Exceptional Progeny/(2*Exceptional Progeny+Normal Progeny)]*100.

### Recombinational History of Diplo-*X* Females

To assess whether recombinant bivalents missegregated in oocytes, the Diplo-*X* female progeny obtained from NDJ tests were genotyped. Each Diplo-*X* female was crossed to two *yw* males and the genotype of the *X* chromosomes of the Diplo-*X* female was inferred from the *sc*, *cv*, *v*, *f*, *car* markers in her male progeny (see Figure 6). Because some fraction of Diplo-*X* females either died before they could be genotyped or were sterile/sub-fertile, the number of Diplo-*X* females genotyped was lower than the number of Diplo-*X* females recovered from the NDJ test.

### Light Microscopic Analysis of Ovaries and Ovarioles

After female flies were subjected to the four-day aging regimen, ovaries from experimental and control females were hand-dissected in modified Robb's buffer [36] at 8 hour intervals and fixed in 4% formaldehyde, PBS (130 mM NaCl, 7 mM Na_2_HPO_4_ and 3 mM NaH_2_PO_4_) for 10 min. The ovaries were rinsed in PBS containing 0.01% Tween-20. Images were captured using a SMZ1500 stereo-microscope (Nikon) equipped with a Pixelink camera (Diagnostic Instruments).

To quantify the relative number of ooctyes at specific developmental stages, a single fixed ovary from three females was teased to generate individual ovarioles. Oocyte stages were tabulated and averaged for aged and non-aged ovaries at each time point. Oocytes were staged based on the standard morphological criteria described by Mahowald and Kambysellis [43]. Briefly, oocyte stages were distinguished based on the size of the egg chamber, the relative size of the oocyte and the presence of yolk within the oocyte. Yolk deposition begins during stage 8 and provides a convenient marker. Mature (stage 13–14) oocytes were distinguished by the presence of chorionic appendages.

### Immunoblot Analysis of SMC1 Levels in Ovary Extracts

Newly eclosed wild-type and *smc1* heterozygous females were fattened with yeast for two days. 30 sets of ovaries from each genotype were dissected in modified Robb's buffer [36] and frozen in liquid N_2_. Each frozen tissue sample was homogenized in 240 µL of buffer containing 8 M Urea, 2% SDS, 100 mM Tris HCl pH 6.8 and 5% Ficoll containing 4 mM AEBSF (protease inhibitor, Sigma). The extract was cleared by centrifugation at 13,000 rpm for 10 min at room temperature and the supernatant was aliquoted and frozen in liquid N_2_. The protein concentration for each extract was determined using a Bradford assay (Biorad). For each genotype, 10 and 20 µg of total protein was separated by SDS-PAGE on a 7.5% gel and transferred to PVDF membrane. The blot was cut horizontally and anti-SMC1 guinea pig serum [11] was used at 1∶1000 dilution for the top half of the blot and monoclonal DM1A (Sigma) was used at 1∶15,000 to detect the α-tubulin loading control on the bottom half of the blot.

### Data Analysis and Statistical Tests

To compare the relative frequency of missegregation occurring in aged and non-aged oocytes, an odds ratio (OR) was calculated. The odds ratio provides a method for determining whether the frequency of a certain event (missegregation) is equal for two different treatments (aged and non-aged). In our analysis, it is calculated as: 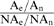 where A_e_ = # of exceptional progeny from aged oocytes, A_n_ = # of normal progeny from aged oocytes, NA_e_ = # of exceptional progeny from non-aged oocytes, and NA_n_ = # of normal progeny from non-aged oocytes [44]. If the treatments result in equal frequencies of the event, then the odds ratio will be 1. In our analysis, an OR >1 implies that the frequency of missegregation is higher when oocytes undergo aging than when they do not undergo aging.

Standard errors (SE) are calculated on the logarithm of the odds ratio according to: 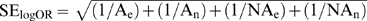. In Figures 5 and 8, error bars represent ±1 SE_logOR_ retransformed back into non-logarithmic units.

For the NDJ tests, *P* values were calculated using a 2×2 χ^2^ Contingency Test (two-tailed). For these calculations, raw data values (not adjusted values) for a specific brood or sub-brood were compared for aged versus non-aged treatments (# of exceptions and # of normal progeny for aged and non-aged). A 2×2 χ^2^ Contingency Test also was used to analyze the recombinational history of missegregating chromosomes recovered in Diplo-*X* progeny. For all tests, a *P* value of <0.05 was considered statistically significant (rejection of the null hypothesis that the two groups are the same).

## Supporting Information

Figure S1 Excess of mature oocytes in the aged ovaries is cleared by 16 hours after the aging regimen. This figure shows representative images of whole ovaries and single ovarioles from *smc1* ^+/−^ *mtrm* ^+/−^ control and experimental females dissected at 8-hour intervals after the aging regimen. Mature oocytes (stage13/14) at the posterior end of the ovariole can be distinguished (in part) by their chorionic appendages (yellow arrows). The scale bars are 100 microns. At time-point 0 after the aging regimen, mature oocytes are far more abundant in the aged ovary than in the non-aged ovary. It is not uncommon to observe two mature oocytes per ovariole in aged ovaries at this time-point. When both experimental and control females are allowed to lay eggs for 8 hours after the regimen, the difference between the number of mature oocytes in the aged ovary and non-aged ovary is reduced. By 16 hours, the excess mature oocytes have been cleared from the aged ovaries, and the distribution of stages found in experimental and control females is very similar (see Figure 7).
(9.77 MB TIF)

Figure S2 Non-recombinant chromosomes exhibit increased missegregation when oocytes undergo aging. The number of non-recombinant (NR) Diplos recovered in sub-broods 1, 3, 4 and 6 is shown for aged oocytes and non-aged oocytes (also see data in Tables S6 & S7). The frequency of NR Diplos (NR/N where N is total progeny) is similar for all four sub-broods that arise from non-aged oocytes (0.72<*P*<0.76 for pair-wise comparisons). Non-recombinant chromosomes in aged oocytes missegregate more frequently in sub-broods 3, 4 and 6 than in sub-brood 1, although this difference is not statistically significant (0.10<P<0.27). However, a comparison of sub-brood 3 aged and non-aged oocytes shows that aging causes a significant increase in the frequency at which non-recombinant chromosomes missegregate (*P* = 0.029). These data reinforce the model that the achiasmate chromosome segregation pathway is vulnerable to age effects and that sister-chromatid cohesion proteins play a role in this process (see Discussion).
(0.18 MB TIF)

Table S1 *X* chromosome nondisjunction in *smc1^+/−^ and mtrm^+/−^*oocytes (no aging regimen).
(0.03 MB DOC)

Table S2 Higher meiotic missegregation is not observed in *smc1^+/−^* aged oocytes when the achiasmate pathway is functional.
(0.03 MB DOC)

Table S3 Age-dependent segregation errors arise in *smc1^+/−^* oocytes when achiasmate segregation also is disrupted.
(0.07 MB DOC)

Table S4 Segregation errors do not increase with age when the achiasmate pathway is compromised but cohesion is wild type.
(0.04 MB DOC)

Table S5 Diplo-*X* females arise from missegregation of both recombinant and non-recombinant chromosomes in aged and non-aged *smc1^+/−^ mtrm^+/−^* oocytes.
(0.06 MB DOC)

Table S6 *smc1^+/−^ mtrm^+/−^* oocytes that give rise to sub-broods 3 and 4 are prone to age-dependent NDJ.
(0.06 MB DOC)

Table S7 Genotype of Diplo-*X* females arising from aged *smc1^+/−^ mtrm^+/−^* oocytes.
(0.07 MB DOC)

Table S8 Genotype of Diplo-*X* females arising from non-aged *smc1^+/−^ mtrm^+/−^* oocytes.
(0.08 MB DOC)
