# Supplementary figures and images for: Aging Predisposes Oocytes to Meiotic Nondisjunction When the Cohesin Subunit SMC1 Is Reduced
Source: PLoS Genet. 2008 Nov 14;4(11):e1000263. doi: 10.1371/journal.pgen.1000263 (PMC2577922; doi:10.1371/journal.pgen.1000263)

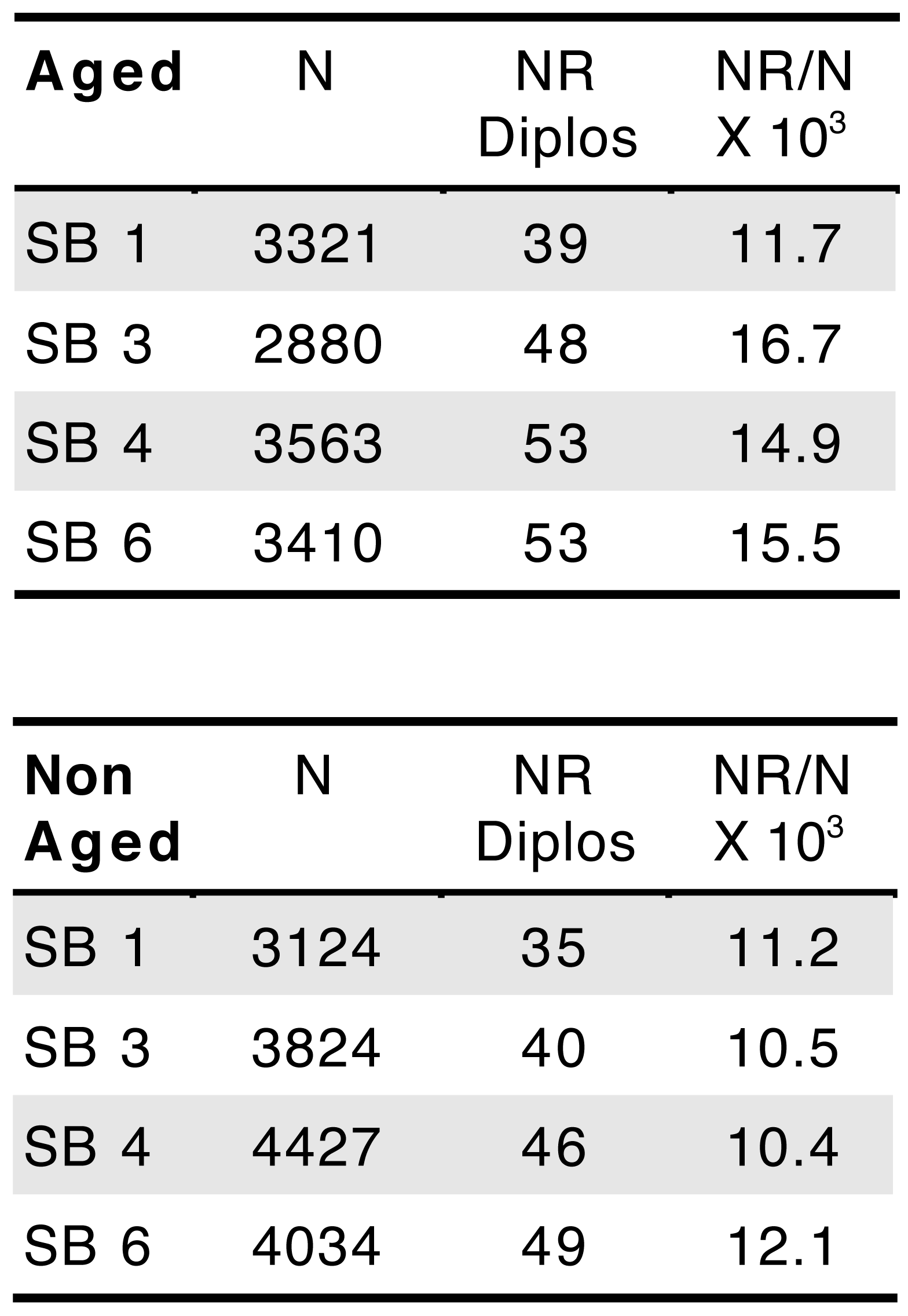

Supplement: Figure S2 — Non-recombinant chromosomes exhibit increased missegregation when oocytes undergo aging. The number of non-recombinant (NR) Diplos recovered in sub-broods 1, 3, 4 and 6 is shown for aged oocytes and non-aged oocytes (also see data in Tables S6 & S7). The frequency of NR Diplos (NR/N where N is total progeny) is similar for all four sub-broods that arise from non-aged oocytes (0.72<P<0.76 for pair-wise comparisons). Non-recombinant chromosomes in aged oocytes missegregate more frequently in sub-broods 3, 4 and 6 than in sub-brood 1, although this difference is not statistically significant (0.10<P<0.27). However, a comparison of sub-brood 3 aged and non-aged oocytes shows that aging causes a significant increase in the frequency at which non-recombinant chromosomes missegregate (P = 0.029). These data reinforce the model that the achiasmate chromosome segregation pathway is vulnerable to age effects and that sister-chromatid cohesion proteins play a role in this process (see Discussion). (0.18 MB TIF) [file pgen.1000263.s002.tif]
