# Supplementary material for: Aging Predisposes Oocytes to Meiotic Nondisjunction When the Cohesin Subunit SMC1 Is Reduced
Source: PLoS Genet. 2008 Nov 14;4(11):e1000263. doi: 10.1371/journal.pgen.1000263 (PMC2577922; doi:10.1371/journal.pgen.1000263)
Supplement: Table S1 — X chromosome nondisjunction in smc1+/− and mtrm+/−oocytes (no aging regimen). (0.03 MB DOC) [file pgen.1000263.s003.doc]

**Table S1:**

***X* chromosome nondisjunction in *smc1^+/-^* and *mtrm^+/-^* oocytes (no aging regimen)**

| **Genotype** | **Normal Gametes** | **Diplo**  **Gametes** | **Nullo Gametes** | **Adjusted Total** | **% NDJ** |
| --- | --- | --- | --- | --- | --- |
| *y/yw;+;smc1^+/-^* | 707 | 0 | 0 | 707 | 0.00 |
| *y/yw;+;mtrm^+/-^* | 971 | 14 | 8 | 1015 | 4.33 |
| *In(1)dl-49/y;+;mtrm^+/-^* | 2020 | 139 | 88 | 2474 | 18.35 |
