# Supplementary material for: Aging Predisposes Oocytes to Meiotic Nondisjunction When the Cohesin Subunit SMC1 Is Reduced
Source: PLoS Genet. 2008 Nov 14;4(11):e1000263. doi: 10.1371/journal.pgen.1000263 (PMC2577922; doi:10.1371/journal.pgen.1000263)
Supplement: Table S2 — Higher meiotic missegregation is not observed in smc1+/− aged oocytes when the achiasmate pathway is functional. (0.03 MB DOC) [file pgen.1000263.s004.doc]

**Table S2:**

**Higher meiotic missegregation is not observed in *smc1^+/-^* aged oocytes when the achiasmate pathway is functional**

Genotype: *y/yw;+;smc1^+/-^*

| **24 hour Broods** | **Normal Gametes** | **Diplo**  **Gametes** | **Nullo Gametes** | **Adjusted Total** | **% NDJ** | ***P* value** |
| --- | --- | --- | --- | --- | --- | --- |
| Aged-1 | 2898 | 3 | 4 | 2912 | 0.48 | 0.0424 |
| Nonaged-1 | 1953 | 4 | 8 | 1977 | 1.21 |  |
| Aged-2 | 2097 | 0 | 1 | 2099 | 0.10 | 0.4340 |
| Nonaged-2 | 1661 | 1 | 1 | 1665 | 0.24 |  |
