# Supplementary material for: Aging Predisposes Oocytes to Meiotic Nondisjunction When the Cohesin Subunit SMC1 Is Reduced
Source: PLoS Genet. 2008 Nov 14;4(11):e1000263. doi: 10.1371/journal.pgen.1000263 (PMC2577922; doi:10.1371/journal.pgen.1000263)
Supplement: Table S3 — Age-dependent segregation errors arise in smc1+/− oocytes when achiasmate segregation also is disrupted. (0.07 MB DOC) [file pgen.1000263.s005.doc]

**Table S3A:**

**Age-dependent segregation errors arise in *smc1^+/-^* oocytes when achiasmate segregation also is disrupted**

Genotype: *y sc cv v f car/y;+;smc1^+/-^ mtrm^+/-^*

| **24 hour Broods** | **Normal Gametes** | **Diplo**  **Gametes** | **Nullo Gametes** | **Adjusted Total** | **% NDJ** | ***P* value** |
| --- | --- | --- | --- | --- | --- | --- |
| Aged-1 | 1171 | 31 | 19 | 1271 | 7.87 | 0.0010 |
| Nonaged-1 | 1101 | 13 | 7 | 1141 | 3.51 |  |
| Aged-2 | 1196 | 30 | 23 | 1302 | 8.14 | 0.0062 |
| Nonaged-2 | 1367 | 20 | 13 | 1433 | 4.61 |  |
| Aged-3 | 1060 | 18 | 7 | 1110 | 4.50 | 0.4751 |
| Nonaged-3 | 1397 | 14 | 13 | 1451 | 3.72 |  |

This table is represented in Fig 5A, Experiment I

**Table S3B:**

**Age-dependent segregation errors arise in *smc1^+/-^* oocytes when achiasmate segregation also is disrupted**

Genotype: *y sc cv v f car/y;+;smc1^+/-^ mtrm^+/-^*

| **24 hour Broods** | **Normal Gametes** | **Diplo**  **Gametes** | **Nullo Gametes** | **Adjusted Total** | **% NDJ** | ***P* value** |
| --- | --- | --- | --- | --- | --- | --- |
| Aged-1 | 2089 | 52 | 38 | 2269 | 7.93 | 0.0017 |
| Nonaged-1 | 1687 | 26 | 14 | 1767 | 4.53 |  |
| Aged-2 | 1495 | 39 | 13 | 1599 | 6.50 | 0.0439 |
| Nonaged-2 | 1254 | 22 | 5 | 1308 | 4.13 |  |
| Aged-3 | 1431 | 22 | 11 | 1497 | 4.41 | 0.1416 |
| Nonaged-3 | 1588 | 32 | 19 | 1690 | 6.04 |  |

This table is represented in Fig 5A, Experiment II
