# Supplementary material for: Aging Predisposes Oocytes to Meiotic Nondisjunction When the Cohesin Subunit SMC1 Is Reduced
Source: PLoS Genet. 2008 Nov 14;4(11):e1000263. doi: 10.1371/journal.pgen.1000263 (PMC2577922; doi:10.1371/journal.pgen.1000263)
Supplement: Table S4 — Segregation errors do not increase with age when the achiasmate pathway is compromised but cohesion is wild type. (0.04 MB DOC) [file pgen.1000263.s006.doc]

**Table S4:**

**Segregation errors do not increase with age when the achiasmate pathway is compromised but cohesion is wild type**

Genotype: *y/yw;+;mtrm^+/-^*

| **24 hour Broods** | **Normal Gametes** | **Diplo**  **Gametes** | **Nullo Gametes** | **Adjusted Total** | **% NDJ** | ***P* value** |
| --- | --- | --- | --- | --- | --- | --- |
| Aged-1 | 3853 | 34 | 30 | 3981 | 3.22 | 0.4543 |
| Nonaged-1 | 3916 | 47 | 27 | 4064 | 3.64 |  |
| Aged-2 | 3371 | 33 | 17 | 3471 | 2.88 | 0.0123 |
| Nonaged-2 | 3886 | 24 | 9 | 3952 | 1.67 |  |
| Aged-3 | 3450 | 14 | 10 | 3498 | 1.37 | 0.8875 |
| Nonaged-3 | 3609 | 19 | 7 | 3661 | 1.42 |  |
