# Supplementary material for: Aging Predisposes Oocytes to Meiotic Nondisjunction When the Cohesin Subunit SMC1 Is Reduced
Source: PLoS Genet. 2008 Nov 14;4(11):e1000263. doi: 10.1371/journal.pgen.1000263 (PMC2577922; doi:10.1371/journal.pgen.1000263)
Supplement: Table S5 — Diplo-X females arise from missegregation of both recombinant and non-recombinant chromosomes in aged and non-aged smc1+/− mtrm+/− oocytes. (0.06 MB DOC) [file pgen.1000263.s007.doc]

**Table S5:**

**Diplo-*X* females arise from missegregation of both recombinant and non-recombinant chromosomes in aged and non-aged *smc1^+/-^ mtrm^+/-^* oocytes**

|  | **Genotype of Diplo *XX* female** | **Aged-1** | **Nonaged-1** |
| --- | --- | --- | --- |
| +/- | y + + + + + / y sc cv v f car | 32 | 16 |
| +/+ | y + + + + + / y + + + + + | 1 | 0 |
| A/+ | y + cv v f car/ y + + + + + | 4 | 0 |
| A/A | y sc + + + + / y + cv v f car | 0 | 1 |
| B/+ | y + + v f car/ y + + + + + | 2 | 0 |
| B/- | y sc cv + + + / y sc cv v f car | 1 | 0 |
| B/B | y sc cv + + + / y + + v f car | 2 | 1 |
| B/+ | y sc cv + + + / y + + + + + | 1 | 0 |
| B/AB | y + + v f car/ y + cv + + + | 1 | 0 |
| C/+ | y + + + f car/ y + + + + + | 0 | 2 |
| C/- | y sc cv v + + / y sc cv v f car | 1 | 1 |
| C/C | y sc cv v + + / y + + + f car | 3 | 1 |
| AC/+ | y sc + + f car/ y + + + + + | 0 | 1 |
| BC/- | y + + v + + / y sc cv v f car | 1 | 0 |

“y + + + + +” and “y sc cv v f car” represent non-recombinant chromosomes.

The left column depicts crossovers observed in the following intervals:

A= sc-cv; B= cv-v; C= v-f; D=f-car

Diplo-*X* females in this table are derived from brood 1 oocytes represented in Table S3.
