# Supplementary material for: Aging Predisposes Oocytes to Meiotic Nondisjunction When the Cohesin Subunit SMC1 Is Reduced
Source: PLoS Genet. 2008 Nov 14;4(11):e1000263. doi: 10.1371/journal.pgen.1000263 (PMC2577922; doi:10.1371/journal.pgen.1000263)
Supplement: Table S6 — smc1+/− mtrm+/− oocytes that give rise to sub-broods 3 and 4 are prone to age-dependent NDJ. (0.06 MB DOC) [file pgen.1000263.s008.doc]

**Table S6:**

***smc1^+/-^ mtrm^+/-^* oocytes that give rise to sub-broods 3 and 4 are prone to age-dependent NDJ**

Genotype: *y sc cv v f car/y;+;smc1^+/-^ mtrm^+/-^*

| **8-hour Broods** | **Normal Gametes** | **Diplo**  **Gametes** | **Nullo Gametes** | **Adjusted Total** | **% NDJ** | ***P* value** |
| --- | --- | --- | --- | --- | --- | --- |
| Aged-1 | 3151 | 85 | 85 | 3491 | 9.74 | 0.7773 |
| Nonaged-1 | 2969 | 77 | 78 | 3279 | 9.45 |  |
| Aged-2 | 2475 | 62 | 77 | 2753 | 10.10 | 0.0614 |
| Nonaged-2 | 3325 | 82 | 67 | 3623 | 8.23 |  |
| Aged-3 | 2705 | 98 | 77 | 3055 | 11.46 | 0.0008 |
| Nonaged-3 | 3661 | 83 | 80 | 3987 | 8.18 |  |
| Aged-4 | 3380 | 91 | 92 | 3746 | 9.77 | 0.0031 |
| Nonaged-4 | 4260 | 86 | 81 | 4594 | 7.27 |  |
| Aged-5 | 3356 | 93 | 94 | 3730 | 10.03 | 0.5770 |
| Nonaged-5 | 3559 | 83 | 104 | 3933 | 9.51 |  |
| Aged-6 | 3265 | 77 | 68 | 3555 | 8.16 | 0.8875 |
| Nonaged-6 | 3865 | 90 | 79 | 4203 | 8.04 |  |

Combined data from two independent experiments
