# Supplementary material for: Aging Predisposes Oocytes to Meiotic Nondisjunction When the Cohesin Subunit SMC1 Is Reduced
Source: PLoS Genet. 2008 Nov 14;4(11):e1000263. doi: 10.1371/journal.pgen.1000263 (PMC2577922; doi:10.1371/journal.pgen.1000263)
Supplement: Table S8 — Genotype of Diplo-X females arising from non-aged smc1+/− mtrm+/− oocytes. (0.08 MB DOC) [file pgen.1000263.s010.doc]

**Table S8:**

**Genotype of Diplo-*X* females arising from non-aged *smc1^+/-^ mtrm^+/-^* oocytes**

|  | **Genotype of Diplo *XX* female** | **Non-aged oocytes** | | | |
| --- | --- | --- | --- | --- | --- |
|  |  | **SB-1** | **SB-3** | **SB-4** | **SB-6** |
| +/- | y + + + + + / y sc cv v f car | 32 | 40 | 44 | 46 |
| +/+ | y + + + + + / y + + + + + | 0 | 0 | 2 | 2 |
| -/- | y sc cv v f car/ y sc cv v f car | 3 | 0 | 0 | 1 |
| A/+ | y + cv v f car/ y + + + + + | 1 | 1 | 1 | 1 |
| A/- | y sc + + + + / y sc cv v f car | 1 | 2 | 1 | 1 |
| A/A | y sc + + + + / y + cv v f car | 1 | 3 | 3 | 2 |
| B/+ | y + + v f car/ y + + + + + | 1 | 3 | 7 | 3 |
| B/- | y sc cv + + + / y sc cv v f car | 3 | 3 | 2 | 3 |
| B/B | y sc cv + + + / y + + v f car | 3 | 3 | 2 | 5 |
| AB/B | y sc + v f car/ y + + v f car | 1 | 0 | 0 | 0 |
| C/+ | y + + + f car/ y + + + + + | 4 | 0 | 3 | 3 |
| C/- | y sc cv v + + / y sc cv v f car | 0 | 1 | 3 | 1 |
| C/C | y sc cv v + + / y + + + f car | 2 | 7 | 2 | 3 |
| B/C | y + + v f car/ y sc cv v + + | 1 | 1 | 0 | 0 |
| B/C | y sc cv + + + / y + + + f car | 0 | 0 | 1 | 1 |
| AC/C | y + cv v + + / y + + + f car | 0 | 0 | 1 | 0 |
| AC/AC | y + cv v + + / y sc + + f car | 0 | 0 | 1 | 0 |
| BC/+ | y sc cv + f car/ y + + + + + | 0 | 0 | 1 | 0 |
| BC/BC | y sc cv + f car/ y + + v + + | 0 | 0 | 0 | 1 |
| D/+ | y + + + + car/ y + + + + + | 3 | 4 | 0 | 0 |
| D/D | y + + + + car/ y sc cv v f + | 0 | 1 | 0 | 1 |
| BD/D | y + + v f + / y + + + + car | 0 | 0 | 1 | 0 |
| BD/BD | y + + v f + / y sc cv + + car | 0 | 0 | 0 | 1 |
| CD/+ | y sc cv v + car/ y + + + + + | 0 | 0 | 1 | 0 |
| A/ABD | y + cv v f car/ y sc + v f + | 0 | 0 | 0 | 1 |
| Other R Diplos |  | 3 | 2 | 1 | 3 |

SB= sub-brood; SB-1: 0-8hrs, SB-3: 16-24 hrs, SB-4: 24-32 hrs, SB-6: 40-48 hrs

“y + + + + +” and “y sc cv v f car” represent non-recombinant chromosomes.

The left column depicts crossovers observed in the following intervals:

A= sc-cv; B= cv-v; C= v-f; D=f-car

Diplo-*X* females shown are from “non-aged” sub-broods 1, 3, 4 and 6 in Table S6.
